# Leiomyosarcome de la langue: à propos d'un cas

**DOI:** 10.11604/pamj.2015.22.8.5208

**Published:** 2015-09-04

**Authors:** Lahcen El jahd, Ismail Barhmi, Nabil Tazi, Sami Rouadi, Reda Abada, Mohammed Roubal, Abdelaziz janah, Mohammed Mahtar

**Affiliations:** 1 Service d'ORL et Chirurgie Cervico-Faciale, Hôpital 20 Août 1953, CHU Ibn Rochd, Casablanca, Maroc

**Keywords:** Léiomyosarcome, langue, muscle lisse, Leiomyosarcoma, tongue, smooth muscle

## Abstract

Le léiomyosarcome primitif de la langue est une tumeur rare qui se développe aux dépens des fibres musculaires lisses. Le diagnostic est souvent difficile, fondé sur des caractéristiques immuno-histologiques particulières. L'objectif de ce travail est de décrire le profil épidémiologique, clinique, thérapeutique et évolutif du léiomyosarcome à travers un cas et une revue de la littérature. Nous rapportons le cas d'un homme âgé de 26 ans, sans antécédents pathologique particuliers, consultant pour une tuméfaction de la langue mobile évoluant depuis 2 ans. Une biopsie de la masse a été réalisée. L’étude anatomopathologique et immunohistochimique a confirmé le diagnostic d'un léiomyosarcome de la langue. L'IRM de la langue a objectivé un processus lesionnel intéressant la portion mobile et antérieur de la langue. Une exérèse de la masse a été réalisée. L'examen histologique a montré la présence d'un large néoplasme de 6 cm compatible à un léiomyosarcome peu différencié de la langue, de garde II selon la Fédération Nationale des Centres de Lutte Contre le Cancer (FNCLCC). Une radiothérapie externe sur la cavité buccale avec une dose de 65 Gy a été réalisée. Le patient a présenté 2 mois après la fin du traitement une adénopathie latéro-cervicale haute gauche (territoire II), il a bénéficié d'un curage ganglionnaire fonctionnel intéressant les territoires I, II et III puis réadressé en radiothérapie. Le léiomyosarcome de la langue est très rare surtout chez le sujet jeune. La chirurgie et la radiothérapie sont les armes thérapeutiques majeures. Le pronostic est très mauvais, Les facteurs les plus importants sont les marges d'exérèse et le grade.

## Introduction

Les Sarcomes des tissus mous représentent 0,7% de toutes les tumeurs malignes, et le léiomyosarcome représente 3% à 7% des sarcomes des tissus mous [1]. Il s'agit d'une tumeur maligne des muscles lisses. Les sites les plus fréquents sont le myomètre utérin et le tractus gastro-intestinal. Sa localisation cervicofaciale est rare, (1% des sarcomes avant 20 ans) [2, 3], ceci est dû à la rareté du tissu musculaire lisse dans cette région. Dans la cavité orale les leiomyosarcomes ont généralement comme point de depart la muqueuse buccale, la gencive et le plancher buccal. La localisation linguale est très exceptionnelle, seulement quelques cas dans la littérature sont rapportés [4, 5].

## Patient et observation

**Figure 1:**
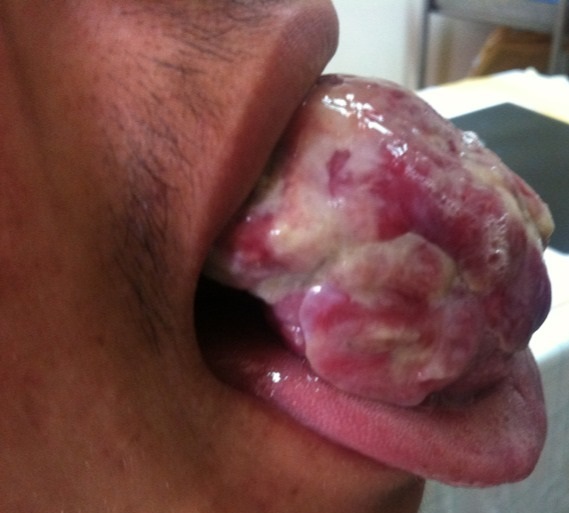
Énorme masse de la portion mobile de la langue

Il s'agit d'un patient âgé de 26 ans, sans antécédents pathologique particuliers, consultant pour une tuméfaction la portion mobile de la langue évoluant depuis 2 ans dans un contexte de conservation de l’état general (Figure 1).

### Examen clinique

**Figure 2:**
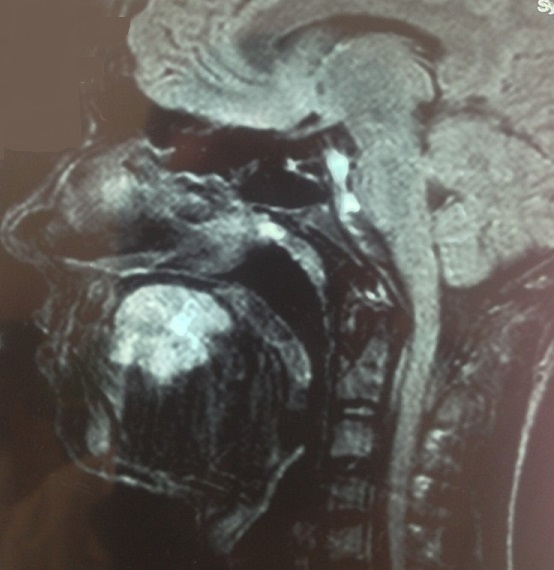
IRM de la langue objective un processus expansif hétérogène intéressant la portion antérieure de la langue, avec une prise de contraste importante

**Figure 3:**
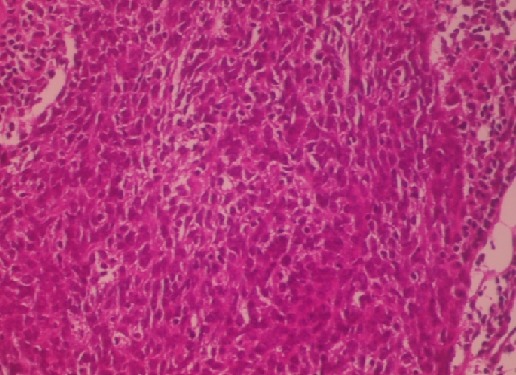
Coupe histologique montrant des cellules fusiformes sarcomateuses

**Figure 4:**
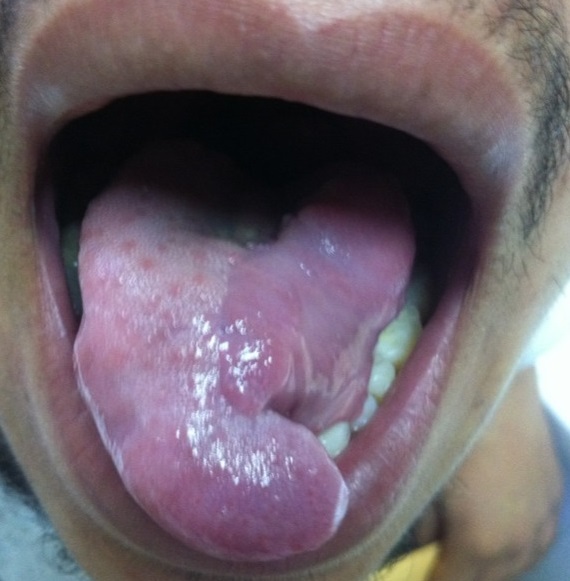
Exérèse de la masse, aspect post opératoire du patient

Masse de la portion mobile de la langue empêchant la fermeture de la cavité buccale, indolore et non hémorragique. La base de langue était souple.les aires ganglionnaires étaient libres. L'IRM de la langue a objectivé un processus expansif hétérogène intéressant la portion mobile et antérieure de la langue(Figure 2). La lésion présente un aspect hypointense en T1et hyperintense en T2 et en T2 FAT SAT. L'injection de gadolinium entraine une prise de contraste hétérogène mais intense de la lésion, La base de la lange et saine sans extension vers l'oropharynx, ni le rhinopharynx. Une biopsie de la masse a été réalisée. L’étude anatomopathologique a trouvé des cellules fusiformes, sarcomateuses, évoquant soit un leiomyosarome soit un rabdomyosarcome. Le complément immuno-histochimique a montré que ces cellules expriment fortement la H-caldesmone, la desmine et l'actine muscle lisse (Figure 3) ce qui confirme un leiomyosarcome de la langue. Le bilan d'extension notamment le scanner thoracique était normal. Une exérèse de la masse a été réalisée (Figure 4). L′examen histologique de la pièce opératoire a montré la présence d'un large néoplasme de 6 cm compatible à un léiomyosarcome peu différencié de garde II selon FNCLCC de la langue avec des marges d'exérèses saines (0,5cm 0,8cm 0,6 cm et 0,5cm) et index mitotique faible (5 à 8 miotses/chps/10 chps). Une radiothérapie externe sur la cavité buccale avec une dose de 65 Gy a été réalisée.

**Figure 5:**
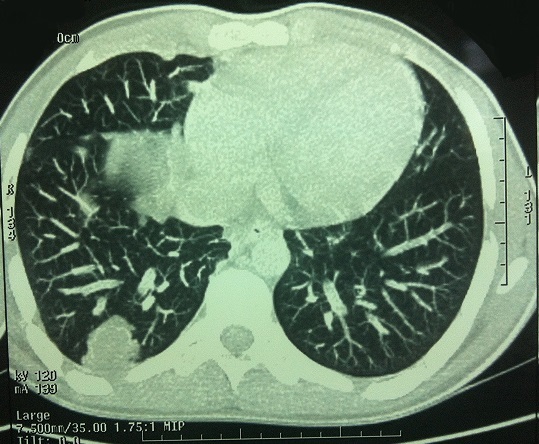
TDM thoracique montre une lésion bilobée de la partie postérieure du segment postéro-basal du lobe inférieur du poumon droit

Le patient a présenté 2 mois après la fin du traitement une adénopathie latéro-cervicale gauche haute (territoire II), pour laquelle il a bénéficié d'un curage ganglionnaire fonctionnel bilatéral intéressant les territoires I, II et III. L’étude anatomopathologique a trouvé un seul ganglion métastatique, avec rupture capsulaire au niveau rétrospinal gauche. réadresser en radiothérapie ou il a bénéficié d'un autre bilan d'extension à base de TDM thoraco-abdominal qui a montré la présence de deux lésions nodulaires intéressant la partie antérieure du lobe supérieur droit mesurée à 7,4mm ainsi qu'une lésion bilobée de la partie postérieure du segment postéro-basal du lobe inférieur mesurée à 15,3mm évoquant des localisations secondaires (Figure 5). Après une concertation multidisciplinaire une chimiothérapie adjuvante a été décidée.

## Discussion

Les Sarcomes des tissus mous de la tête et du cou sont rares chez les adultes, probablement à cause de la rareté du tissu musculaire lisse dans ces sites [6]. Ils représentent environ 1% de tous les cancers de la tête et du cou et 7% de tous les sarcomes des tissus mous [7]. Ils peuvent survenir dans n′importe quel endroit, mais plus de la moitié sont situés dans les sites rétropéritonéaux ou intra-abdominale. Le léïomyosarcome survient chez l'adulte, avec une moyenne d’âge de 58 ans [7], La séméiologie clinique est banale, manifestée par une masse indolore, circonscrite et fortement adhérente aux muscles linguales [8].

Le diagnostic est confirmé par une étude histologique réalisée à la suite d'une biopsie. Le diagnostic anatomopathologique, complété par une étude immuno-histochimique est indispensable pour la classification de ces tumeurs. Les Léiomyosarcome sont classées en trois stades selon leur différenciation, l'activité mitotique et la nécrose. La majorité des leiomyosarcomes sont hypercellulaires, de haut grade, composés de cellules fusiformes éosinophiles avec une activité mitotique accrue et une atypie nucléaire sévère à modérée. Le Leiomyosarcome de bas grade, avec atypie modérée, et des mitoses dispersées est très rare. Les cellules néoplasiques expriment généralement la vimentine, l'actine musculaire lisse et la desmine, tandis que l'expression de la cytokératine n'est observée que dans 25% des cas [9–12].

L'IRM est l'examen de choix permet d’évaluer l′étendue de la lésion primaire d’évaluer la base de la langue, le plancher buccal et les ganglions lymphatiques régionaux. La TDM joue un rôle fondamental, dans l’évaluation de l'extension tumorale à distance au niveau du foie, poumon, os et ganglions, et permet l'estimation de la réponse thérapeutique. Généralement les poumons sont les sites les plus communs de diffusion métastatique suivie par le foie et l'os, La probabilité de métastases ganglionnaires régionales est faible [13].

Le traitement de cette tumeur n'est pas encore codifié, car les cas sont rare, il inclut chirurgie, radiothérapie pré ou post-opératoire et chimiothérapie néo-adjuvante ou adjuvante selon les cas. Il dépend de l’âge, de l’état général, du volume tumoral, du grade de malignité et du bilan d'extension [14]. Le traitement optimal est la résection complète avec des marges de résection de 1cm. Le curage ganglionnaire est réservé aux cas avec atteinte ganglionnaire régionale. La radiothérapie post opératoire adjuvante est indiquée pour les patients atteints de bas grade tumoral avec des marges d'exérèses inférieures à 1cm, ainsi que ceux de haut grade ou en cas d'envahissement ganglionnaire histologique. Cette radiothérapie est souvent associée à une chimiothérapie à base de cisplatine et de métotrexate.Si la chirurgie n′est pas possible, la tumeur est traitée avec radiothérapie définitive à une dose élevée (74,4 à 81,6 Gy à 1,2 Gy par fraction deux fois par jour dans un cours continu). Il est difficile d′évaluer l′efficacité de la radiothérapie seule, car elle est généralement utilisée pour les grandes tumeurs non résécables qui ont une faible chance de guérison après une modalité de traitement [15]. Cependant, il est probable que la chirurgie et radiothérapie sont plus efficaces que la radiothérapie seule pour les tumeurs résécables [16].

Le pronostic des leïomyosarcomes de la langue est souvent défavorable La survie est très variable selon les séries, elle est en moyenne de moins de 10% à 5 ans le plus souvent à cause de métastases surtout pulmonaires (40%) et hépatiques. Le taux de récidive est élevé [17], justifiant une surveillance régulière d'au moins cinq ans.

## Conclusion

Le léïomyosarcome de la langue est une tumeur rare, La TDM et surtout l'IRM jouent un rôle important dans l’évaluation de la tumeur dans le bilan d'extension et le suivi post-thérapeutique, mais seul l'examen anatomopathologique, complété par une étude immuno-histochimique permettent de confirmer le diagnostic. Leur prise en charge thérapeutique n'est pas codifiée à l'heure actuelle et leur pronostic demeure très sombre et ne peut être amélioré qu'avec une approche multi-disciplinaire et un diagnostic précoce, permettant de réaliser une chirurgie radicale complète, seule thérapeutique efficace.
